# ASIAN OLDER ADULTS’ ONLINE AND OFFLINE HEALTH INFORMATION PREFERENCES AND BEHAVIORS

**DOI:** 10.1093/geroni/igz038.1535

**Published:** 2019-11-08

**Authors:** Bo Xie, Kristina Shiroma

**Affiliations:** The University of Texas at Austin, Austin, Texas, United States

## Abstract

Older adults living in Asia or of Asian origin have unique preferences for information that require special attention. This symposium focuses on the health information preferences and behaviors of Asian older adults. Song et al. investigated the relationship between Internet use and perceived loneliness among Older Chinese using from survey data collected in the 2015 wave of the China Health and Retirement Longitudinal Study (CHARLS), a national study involving 12,400 households in Mainland China. Multiple regression results suggest that older Chinese Internet users perceived significantly less loneliness compared with their age peers who were non-Internet users. Zhang et al. investigated the role of information and communication technologies in supporting antiretroviral therapy (ART)-related knowledge seeking among older Chinese with HIV. Their cross-sectional survey data were collected from 2012 to 2013 in Guangxi, China. The results suggest that less than 5% of the participants sought HIV-related information via computers. Patients less knowledgeable about ART were more likely than those more knowledgeable to consult medical professionals about the disease via cell phones. Shiroma et al. report findings of a systematic literature review conducted in spring 2019 that examined Asian ethnic minority older adults’ preferences for end-of-Life (EOL) information seeking and decision making. The results suggest Asian ethnic minority older adults are understudied in the literature on EOL information and decision making, especially in terms of their unique cultural contexts. Du et al. examined how health information obtained from different types of social networks affect osteoporosis self-management behaviors among older White and Asian women.

